# Social Media Influencers and Their Impact on Society in Performing Cosmetic Procedures Among Al-Ahsa Community

**DOI:** 10.7759/cureus.68374

**Published:** 2024-09-01

**Authors:** Latifah A Albash, Tareq Alyahya, Shahd S Albooshal, Sarah A Busbait, Ahmed K Alkhateeb, Batool Y Alturaiki

**Affiliations:** 1 Medicine and Surgery, King Faisal University, Al-Ahsa, SAU; 2 Plastic Surgery, King Faisal University, Al-Ahsa, SAU; 3 Medicine, King Faisal University, Al-Ahsa, SAU; 4 Aesthetic, King Faisal University, Al-Ahsa, SAU

**Keywords:** trend, surgery, social media influencers, social media, cosmetic procedures

## Abstract

Introduction: The popularity of cosmetic surgery has been increasing in recent years, and social media is playing a role in this trend. This study investigated the influence of following social media influencers on the decision to undergo cosmetic procedures in Al Ahsa, Saudi Arabia.

Methodology: A descriptive cross-sectional study was conducted in Al Ahsa, Saudi Arabia. A self-administered questionnaire was used to collect data. Males and females over 18 years old in Al Ahsa were included, while people under 18 years old who did not complete the questionnaire and did not live in Al Ahsa were excluded.

Result: The study found that social media influencers may be influencing the decision of some people to undergo cosmetic procedures. 90.4% of participants agreed that undergoing cosmetic procedures is a popular procedure among influencers on social media. 37.9% of participants said that pictures of cosmetic procedures before and after affect their desire to undergo a cosmetic procedure. There were significant differences in the views of male and female participants.

Conclusion: This study found that social media influencers may influence the decision to undergo plastic surgery, especially among young women. Participants who followed influencers were more likely to believe that influencers had undergone plastic surgery and were more likely to be influenced by the changes shown in before-and-after pictures. These findings suggest that social media may be playing a role in increasing the demand for plastic surgery, especially among young people.

## Introduction

Aesthetic procedures have become increasingly popular in the medical field, driven by factors such as the pursuit of perfection, dissatisfaction with body image, and the pervasive influence of social media [1]. These procedures encompass both surgical operations, like rhinoplasty and breast augmentation, and non-invasive treatments, such as chemical peels and botulinum toxin injections. Social media platforms are now widely used for marketing cosmetic procedures globally, making it crucial to understand how they impact individuals' decisions to undergo such operations. A notable phenomenon known as "Snapchat dysmorphia" has emerged, wherein patients desire surgeries that replicate the appearance-altering effects of Snapchat filters. Survey data from the American Academy of Facial Plastic and Reconstructive Surgery revealed that 55% of patients are willing to undergo cosmetic procedures to modify their looks in selfies [2]. 

While there is existing research on the influence of social media on cosmetic surgery, little is known about the specific factors that drive individuals to undergo these procedures after viewing advertisements or publications on social media. Consequently, it is crucial to study the impact of media and advertising on the public's decision-making process regarding cosmetic procedures. Additionally, the lack of local knowledge regarding the influence of media has created a need to establish a baseline understanding of their overall impact.

The study aims to fill this research gap by examining the influence of following social media influencers and other relevant factors on individuals' decisions and desires to undergo cosmetic treatments. Conducting this study in the Al-Ahsa region will contribute to the existing knowledge, increase awareness, and provide clarity on the relationship between social media influencers and their impact on the decision to undergo cosmetic procedures.

## Materials and methods

Study design

Online questionnaires were used to perform a population-based survey as part of the study, which employed a descriptive cross-sectional design. These surveys were disseminated through social media channels in order to investigate the impact of social media influencers on the frequency of cosmetic treatments in the Al-Ahsa community.

Study area and settings

Al-Ahsa, which is located in Saudi Arabia's Eastern Province and serves as a representative metropolitan region for the nation, was the site of the study. Using social media and an online survey platform, the study recruited participants from the broader Al-Ahsa population. This approach ensured a convenient and effective data collection process by using the widespread usage of social media platforms among Saudi Arabian citizens, including WhatsApp, Instagram, Snapchat, and Twitter. Because all questionnaires and surveys were distributed online, participants could complete them whenever convenient for them and from the comfort of their homes. The online mode improved outreach and accessibility, which raised the possibility of getting a representative sample from the target demographic.

Study population

The demographic of interest for the study was people who were 18 years of age or older. Participants had to fulfil the following inclusion criteria in order to be considered for the study: possess Saudi Arabian citizenship, live in Al-Ahsaa, and be at least 18 years old. We excluded non-Saudi citizens and those under the age of 18. The sample size for this study was calculated using the unknown population sample formula with a 95% confidence interval and 0.05. The total number of participants in the study was 385.

The data collection and analysis

A self-administered questionnaire was constructed using the survey to collect primary data and distribute it via social media platforms (WhatsApp, Twitter, and Telegram). The questionnaire was translated into Arabic, and it included three sections. The first section included informed consent. The second section included age, gender, educational level, and marital status. The final set of questions assessed the impact of emulating social media influencers and other relevant variables on the decision to have cosmetic treatments done in the future.

Data were stored using Microsoft Office Excel (2023, Microsoft® Corp., Redmond, WA). Statistical Package for Social Sciences (SPSS) version 28.0 (Armonk, NY: IBM Corp.) was then supplied with the data. Descriptive statistics, including mean, median, standard deviation, and frequencies, were used to examine and assess quantitative variables, as indicated in the figures. For The chi-square test was used to analyze the qualitative data.

Ethical considerations

This research was approved by King Faisal University (KFU-REC-2023-FEB-ETHICS592). We were granted the privacy and confidentiality of the participants.

## Results

The majority of the participants were under 35 years of age. Nearly one-third (n = 118, 30.6%) were under 25 and (n = 100, 26.0%) between 25 and 35 years old. Also, females represent about two-thirds of our sample (n = 258, 67.0%). Regarding educational level (n = 302, 78.4%), the participants had a university degree. Only a few participants (n = 8, 2.1%) had education below the secondary level. In terms of marital status, 56.4% (n = 217) were married, 40.3% (n = 155) were single, and 3.4% (n = 13) were either divorced or widowed (Table 1).

**Table 1:** Personal data of study participants, Al-Ahsa, Saudi Arabia

Personal data	No	%
Age in years		
<25	118	30.6%
25–35	100	26.0%
36–45	60	15.6%
46–55	64	16.6%
>55	43	11.2%
Gender		
Male	127	33.0%
Female	258	67.0%
Educational level		
Below secondary	8	2.1%
Secondary	75	19.5%
University	302	78.4%
Marital status		
Single	155	40.3%
Married	217	56.4%

Among the study participants, 8.6% (n = 33) reported a history of plastic surgery, while 91.4% (n = 352) had no such history. Out of the 33 participants who had undergone plastic surgery, 33.3% (n = 11) had liposuction, 30.3% (n = 10) had facioplasty, 21.2% (n = 7) had body sculpting, and 15.2% (n = 5) had other types of surgery (Table 2).

**Table 2:** History and types of plastic surgery among study participants, Al-Ahsa, Saudi Arabia

Plastic surgery	No	%
Do you have a previous history of plastic surgery?		
Yes	33	8.6%
No	352	91.4%
If yes, type of surgery (n=33)		
Liposuction	11	33.3%
Facioplasty	10	30.3%
Body sculpting	7	21.2%
Others	5	15.2%

Regarding social media use, 14.5% (n = 56) used social media for one to two hours daily; 39.0% (n = 150) used it for three to four hours; and 43.1% (n = 166) for five to six hours. However, only 3.4% (n = 13) used it for over six hours. Additionally, the majority of 307 (79.7%) followed influencers on social media, while 78 (20.3%) did not. Regarding following plastic surgeons or cosmetic clinics, 35.3% (n = 136) reported doing so, whereas 64.7% (n = 249) did not (Table 3).

**Table 3:** Social media frequency and pattern of use among participants, Al-Ahsa, Saudi Arabia

Social media use	No	%
Hours of using social media daily		
1–2 hours	56	14.5%
3–4 hours	150	39.0%
5–6 hours	166	43.1%
>6 hours	13	3.4%
Do you currently follow or have you ever followed influencers on social media?		
Yes	307	79.7%
No	78	20.3%
Do you currently follow or have you ever followed one of the plastic surgeons or cosmetic clinics on social media?		
Yes	136	35.3%
No	249	64.7%

There was no significant gender difference in the influence of pictures of cosmetic procedures before and after on their desire to undergo a cosmetic procedure (n = 41, 32.3%), females (n = 105, 40.7%, p = 0.110). However, a significant difference was found regarding the belief that undergoing cosmetic procedures could increase Snapchat followers, males (n = 48, 37.8%) and females (75, 29.1%, p = 0.049). Additionally, considering cosmetic procedures popular among influencers shows a statistically significant difference between males (n = 109, 85.8%) and females (n = 239, 92.6%, p = 0.033). Lastly, males (n = 16, 12.6%) and females (n = 60, 23.3%) indicated they might be persuaded to undergo a cosmetic procedure through social media advertisements or posts, with a significant gender difference (p = 0.014; Table 4).

**Table 4:** Social media influencers and their impact on society in performing cosmetic procedures by their gender, Al-Ahsa, Saudi Arabia *P < 0.05 (significant)

Social media influence	Gender				p-value
	Male		Female		
	No	%	No	%	
Pictures of cosmetic procedures before and after affect your desire to undergo a cosmetic procedure, whether surgical or otherwise?	41	32.3%	105	40.7%	0.110
Undergoing cosmetic procedures is a method to increase followers on your Snapchat account	48	37.8%	75	29.1%	0.049*
Undergoing cosmetic procedures is a popular procedure among influencers on social media	109	85.8%	239	92.6%	0.033*
You may be persuaded to undergo a cosmetic procedure through advertisements or posts that appear on social media platforms	16	12.6%	60	23.3%	0.014*

Among participants aged ≤35 years, 39.0% (n = 85) reported that pictures of cosmetic procedures before and after affected their desire to undergo such procedures, compared to 36.5% (n = 61) of those aged >35 years, with no significant difference (p = 0.621). For the belief that undergoing cosmetic procedures could increase Snapchat followers, 28.4% (n = 62) of participants aged ≤35 and 36.5% (n = 61) of those aged >35 had this view. However, this difference was not statistically significant (p = 0.092). Regarding the perception that cosmetic procedures are popular among influencers, 89.9% (n = 196) of participants aged ≤35 and 91.0% (n = 152) of those aged >35 agreed, with no significant difference (p = 0.714). However, a significant difference was found in the likelihood of being persuaded to undergo a cosmetic procedure through social media advertisements or posts. In this, 22.9% (n = 50) of participants aged ≤35 and 15.6% (n = 26) of those aged >35 reported this influence (p = 0.049; Table 5).

**Table 5:** Social media influencers and their impact on society in performing cosmetic procedures by their age, Al-Ahsaa, Saudi Arabia *P < 0.05 (significant)

Social media influence	Age in years				p-value
	≤35 years		>35 years		
	No	%	No	%	
Pictures of cosmetic procedures before and after affect your desire to undergo a cosmetic procedure, whether surgical or otherwise?	85	39.0%	61	36.5%	0.621
Undergoing cosmetic procedures is a method to increase followers on your Snapchat account	62	28.4%	61	36.5%	0.092
Undergoing cosmetic procedures is a popular procedure among influencers on social media	196	89.9%	152	91.0%	0.714
You may be persuaded to undergo a cosmetic procedure through advertisements or posts that appear on social media platforms	50	22.9%	26	15.6%	0.049*

**Figure 1:**
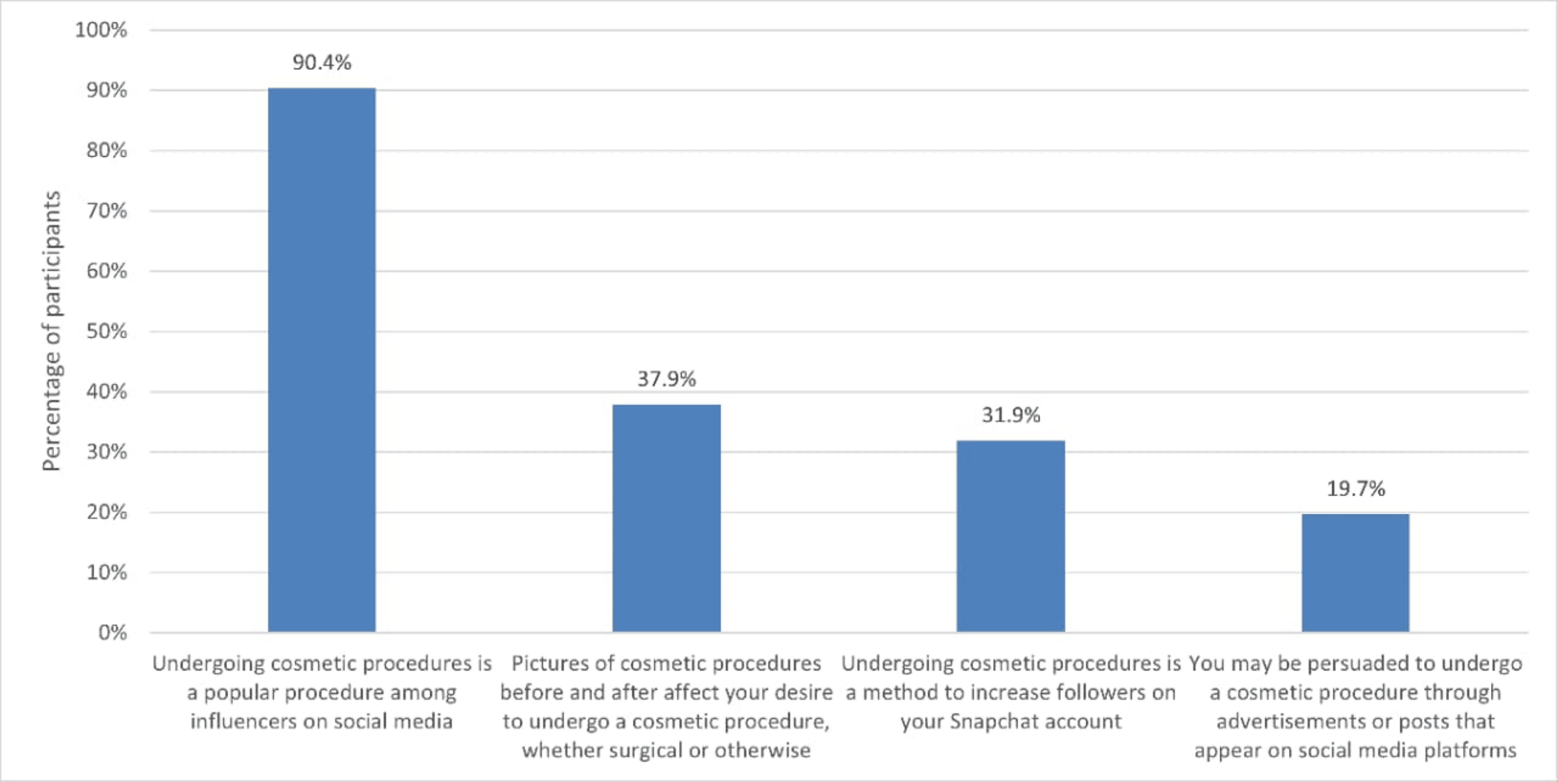
Social media influencers and their impact on society in performing cosmetic procedures among Al-Ahsa community

Also, we analyzed the influence of social media on the desire to undergo cosmetic procedures among the Al-Ahsa community. Almost all the participants (n = 348, 90.4%) agreed that undergoing cosmetic procedures is popular among social media influencers. Additionally, one-third of the participants (n = 146, 37.9%) think that pictures of cosmetic procedures before and after affect their desire to undergo such procedures. About one-third (n = 123, 31.9%) believed that undergoing cosmetic procedures is a method to increase followers on Snapchat. However, only a few (n = 76, 19.7%) indicated that they might be convinced to undergo a cosmetic procedure through advertisements or posts on social media platforms (Figure 1).

## Discussion

Research suggests that spending excessive time on social media and consuming appearance-related content, such as photographs, are associated with greater body image concerns [3]. Multiple studies have shown a link between increased exposure to cosmetic surgery through media and a higher likelihood of undergoing such procedures, particularly among individuals who spend significant amounts of time on social media and follow accounts promoting the benefits of cosmetic surgery [4]. The American Academy of Facial Plastic and Reconstructive Surgery survey also reported increased surgery requests due to social media photo sharing [5]. Similar findings were observed in a recent study conducted in Saudi Arabia, where nearly half of the participants expressed willingness to undergo cosmetic treatments due to social media advertisements [6].

As a significant component of one's identity, one's physical appearance influences how one perceives oneself from an early age [7]. Despite the fact that women's views of attractiveness are driven by natural inclinations such as symmetry and a modest waist-to-hip ratio [8], the sociocultural context can also affect how attractive people are perceived [9]. According to the Tripartite Influence Model [10], peers, parents, and the media are the three main sociocultural influencers that support and spread beauty norms [11].

The current study aimed to assess the impact of social media influencers among the Al-Asha community on the decision to undergo plastic surgery. The study showed that most respondents were young females with a high level of education. A very small percentage (less than 1 out of every 10) had undergone plastic surgery, mainly liposuction and facial procedures. Almasri et al. [12] reported a higher prevalence of cosmetic surgery, finding that about half of the female participants in their study (55.4%) had undergone a cosmetic procedure. Among the participants in the current study, 54.7% of those who had not undergone cosmetic surgery reported not needing it, while 17% cited financial reasons, and 9.4% mentioned social causes as reasons for not undergoing the procedures. Another study by Pempek et al. [13] documented that 10% of Saudi adults had undergone cosmetic surgery, which is similar to the findings of the current study.

Regarding social media use and following social media influencers, the study revealed that nearly half of the participants used social media for at least five hours daily or more. Additionally, most of them currently followed or had previously followed influencers on social media, but only one-third currently followed or had previously followed plastic surgeons or cosmetic clinics on social media. This can be explained by the fact that most participants were young females who had the time to use social media platforms and were mostly influenced by popular social media personalities.

Regarding the effect of social media influencers on participants' decisions towards cosmetic surgery, the current study showed that most participants agreed that undergoing cosmetic procedures is popular among influencers on social media. More than one-third of the participants stated that pictures of cosmetic procedures before and after affected their desire to undergo a cosmetic procedure, whether surgical or non-surgical. About one-third of the participants believed that undergoing cosmetic procedures is a method to increase followers on their Snapchat accounts. Only one-fifth reported that they may be persuaded to undergo a cosmetic procedure through advertisements or posts that appear on social media platforms. These factors were more commonly reported by females than males, except for the belief that cosmetic procedures are a method to increase followers on Snapchat, which was higher among males. Younger participants were also more likely to be persuaded by advertisements or posts on social media platforms. The study showed that only one-third of the participants are influenced by social media and influencers when making decisions about cosmetic surgery, but most of them believe that influencers undergo cosmetic surgery. Pempek et al. [13] reported that social media's visual, picture-oriented nature, mainly Instagram, inspires users to view and comment on the pictures other users display on their profiles. Additionally, de Vries et al. [14] documented that social media may influence many decisions, such as engaging in low-level appearance change, such as dying hair, altering clothes, or changing makeup application.

Brown and Tiggemann [15] noticed that increased social media use has been related to more body image concerns and eating disorders, while Brown and Tiggemann [16] found that viewing pictures of attractive celebrities and peers on Instagram has a negative effect on women's mood and body image. The Royal Society for Public Health [17] reported that Instagram is one of the most detrimental social media platforms for young people's mental health and well-being. The literature also showed that social media may not only change public curiosity levels but may also modify the characteristics of patients seeking these procedures. Instagram is most used by younger adult females and might motivate female patients towards cosmetic clinics seeking these procedures at a younger age, which aligns with the findings of the current study [18-20].

The study had several important limitations that should be recognized. It focused exclusively on Saudi nationals residing in Al-Ahsa City, which restricted the ability to apply the findings to other populations or locations within Saudi Arabia or beyond. Additionally, the use of a cross-sectional design in this research hinders the capability to establish causal correlations. It limits the interpretation of associations between sociodemographic factors and beliefs, awareness, and knowledge.

## Conclusions

In conclusion, the current study showed that very few percent of the study participants had previously undergone plastic surgery, mainly liposuction and facial procedures. Also, about one out of every three participants follows or has ever followed one of the plastic surgeons or cosmetic clinics on social media, but nearly three-fourths follow or have ever followed influencers. The most reported effect of social media influencers was that participants thought that most influencers undergo plastic surgery, and their influence by change was shown by pictures before and after the procedure. Also, social media affected participants' decisions regarding undergoing cosmetic surgery, mainly females, especially through advertisements or posts that appear on social media platforms.

## Appendices

**Table 6:** Questionnaire

Questions	Options
Do you agree to participate in this study?	• Yes • No
Age group	• <25 years • 25–35 years • 36–45 years • 46–55 years • >55 years
Gender	• Male • Female
Educational level	• Primary school • Intermediate school • Secondary school
Marital state	• Single • Married • Divorced • Widowed
Previous history of cosmetic surgery	• Yes • No
Type of plastic surgery	• Liposuction/Tightening • Cosmetic surgery • Face lift blepharoplasty - face contouring • Other
Do the before and after pictures on Snapchat affect your desire for cosmetic intervention, whether surgical or otherwise?	• Yes • No
Is undergoing cosmetic surgery a way to increase followers on your Snapchat account?	• Yes • No
Is undergoing a cosmetic procedure a popular procedure among social media influencers?	• Yes • No
Are you persuaded to undergo a cosmetic surgery from advertisements or publications that appear on the social media platforms?	• Yes • No
